# Treatment of Pharyngitis in Uninsured Patients: A Multicenter Study of Free Clinics

**DOI:** 10.7759/cureus.18564

**Published:** 2021-10-07

**Authors:** Matthew Nguyen, Patrick Dyjak, Madeline MacDonald, Jhulianna Vivar, Shreni Shah, Justin Swanson, Zachary Pruitt, Abu-Sayeef Mirza, Rahul Mhaskar

**Affiliations:** 1 Medicine, University of South Florida Morsani College of Medicine, Allentown, USA; 2 Biomedical Sciences, University of South Florida, Tampa, USA; 3 Internal Medicine, University of South Florida Morsani College of Medicine, Tampa, USA; 4 Biostatistics & Epidemiology, College of Public Health, University of South Florida, Tampa, USA; 5 Public Health Sciences, College of Public Health, University of South Florida, Tampa, USA

**Keywords:** antibiotics, pharyngitis, uninsured, free clinic, tampa bay

## Abstract

Introduction

Appropriate antibiotic prescription practices for pharyngitis slow anti-microbial resistance. Unnecessary antibiotic prescribing and non-adherence to practice guidelines remain a clinical problem. The objective of this study was to examine the relationship between group A Streptococcus (GAS) throat culture testing and antibiotic prescriptions at 10 free clinics in the Tampa Bay Area serving the uninsured population.

Methods

A retrospective cohort study was conducted using data from patient charts from January 2018 to December 2019. We obtained data regarding a chief complaint related to strep pharyngitis: sore throat, enlarged tonsils, pharyngeal erythema, and/or cervical lymphadenopathy. The frequency and relative proportions of throat swab administration and antibiotic prescription were also analyzed.

Results

Of the 12,005 patients serviced during the study period, 245 (2.0%) reported one or more of the chief complaints related to strep pharyngitis. Of the patients reporting pharyngitis, the mean age was 40.2 years, with 66% being female. Of the patients receiving antibiotics for pharyngitis symptoms, 93 (91.2%) did not receive a throat swab. Patients receiving a throat swab showed a significantly increased odds of antibiotic prescription (OR=3.4, 95% CI: 1.1-12.7). Patients reporting symptoms of pharyngitis commonly had other comorbidities, including smoking (14.7%) and diabetes (13.5%).

Conclusion

The large proportion of patients receiving antibiotics for pharyngitis symptoms reveals the need for provider counseling on current recommendations of antibiotic prescription practices, which state that a throat swab with a rapid antigen detection test and/or culture should be performed for all patients where bacterial symptoms of rhinorrhea, cough, and/or oral ulcers are present. Another potential area of improvement indicated by this study may be providing additional supplies of throat swabs for these underserved clinics. Further research is needed to understand the root causes of providers' non-compliant prescribing patterns in the free clinics and to assess the role of the uninsured population in reducing anti-microbial resistance.

## Introduction

Evidence shows that antibiotic over-prescribing practices lead to increased anti-microbial resistance [1]. Over the past two decades, there has been a push to prevent over-prescription of antibiotics. Prescription of antibiotics for viral versus bacterial pharyngitis has been a quality-of-care measure of the National Committee for Quality Assurance (NCQA) since 2004 [2]. Yet, improper antibiotic practices are still prevalent. A 2016 CDC study states that at least 30% of antibiotics prescribed in the outpatient setting were unnecessary [3] and that antibiotic prescription practices should become more tightly controlled. According to the CDC’s 2019 Antibiotic Resistance Threats Report, “more than 2.8 million antibiotic-resistant infections occur in the U.S. each year, and more than 35,000 people die as a result” [4].

One particular area from which to approach the growing threat of anti-microbial resistance is a prescription of antibiotics for acute upper respiratory infections, particularly streptococcal pharyngitis “sore throats.” In 2012, the Infectious Diseases Society of America (IDSA) released guidelines for the diagnosis of group A Streptococcus (GAS) pharyngitis, which state that throat swab along with a rapid antigen detection test (RADT) and/or culture should be performed for all patients where bacterial symptoms, such as rhinorrhea, cough, oral ulcers, and/or ulcers, are present. Following a negative RADT, children and adolescents should receive a back-up throat culture. This culture is not necessary for adults as there is a low incidence of GAS pharyngitis in the adult population [5].

However, many providers do not follow these antibiotic prescription clinical guidelines. In 2006, Machlin and Carper reported that 22% of children with a chief complaint of sore throat were prescribed an antibiotic prescription before receiving a throat swab [6]. A 2018 study reported that more than one-quarter of children with pharyngitis were prescribed antibiotics unnecessarily, indicating a continued gap between treatment recommendations and current practices of a large portion of providers [7]. Luo et al. performed a meta-analysis which found that 57.88% of patients aged 18 years and older were prescribed antibiotics which received no prior testing from 2011 to 2015 [8].

Our study aims to provide a baseline understanding of how antibiotics are being prescribed in free clinics of the Tampa Bay Area.

## Materials and methods

This retrospective cohort study includes all consecutive uninsured patients served at 10 free clinics in the Tampa Bay area in Florida between January 1, 2018, and December 31, 2019. Free clinics are non-profit organizations that rely on volunteer physicians, students, and staff that usually serve vulnerable, low-income, and uninsured/underinsured patients unable to receive care in the usual outpatient setting. Trained undergraduate and medical students conducted a retrospective chart review to identify patients reporting a sore throat, enlarged tonsils, pharyngeal erythema, and/or cervical lymphadenopathy as a major complaint, whether those patients completed a throat swab, and whether those patients were prescribed an antibiotic. We compared demographics (age, sex, race/ethnicity, and employment status) and comorbidities (diabetes, chronic obstructive pulmonary disease [COPD], asthma, bronchitis, smoking, and alcohol consumption) between patients who received a throat swab and patients who did not receive a throat swab. To test differences between these two groups, we used Welch’s t-test for numeric variables and χ^2^ tests for categorical variables; missing values were not included in tests of significance. The association between throat swab completion and antibiotic prescription is presented as an odds ratio (OR) and 95% confidence interval (CI).

Study data were collected and managed using REDCap electronic data capture tools hosted at the University of South Florida [9,10]. Data were analyzed using R statistical software version 4.0.3 (R Foundation for Statistical Computing, Vienna, Austria) [11]. All participating clinics consented to the collection and use of patient data. This study was approved by the University of South Florida Institutional Review Board (Study # Pro00023920).

## Results

During the study period, 12,005 patients were served across 10 clinics. Of these, 245 (2.0%) patients who reported a sore throat, enlarged tonsils, pharyngeal erythema, and/or cervical lymphadenopathy as a major complaint were included for analysis. Of the 245 patients complaining of a sore throat, 102 (41.6%) were prescribed an antibiotic (Table 1). Only nine (8.8%) of the patients prescribed an antibiotic completed a throat swab. Patients receiving a throat swab showed a significantly increased odds of antibiotic prescription (OR=3.4; 95% CI: 1.1-12.7). Patients had a mean age of 40.2 years (standard deviation [SD]=16.8) (Table 2). The majority of patients were female (n=159, 66.0%) and employed (n=46, 57.5%). The largest racial/ethnic groups were non-Hispanic white (n=62, 36.7%) and Hispanic of all races (n=64, 37.9%). Patients who received a throat swab tended to be younger (mean=32.5, SD=15.8) than those who did not receive a throat swab (mean=40.6, SD=16.8), although this difference was non-significant (p=0.093). Significant differences in race/ethnicity were detected between patients with and without a throat swab (p=0.030), with fewer non-Hispanic whites (n=2, 18.2% vs n=60, 38.0%) and Hispanics (n=3, 27.3% vs n=61, 38.6%) among those who did not receive a throat swab and more non-Hispanic blacks (n=5, 45.5% vs n=20, 12.7%) among those who did receive a throat swab. Neither sex (p=0.961) nor employment status (p=0.984) was significantly associated with throat swabs. Throat swabs were not significantly associated with diabetes (p=0.835), COPD (p=0.907), asthma (p=0.993), bronchitis (p=0.725), smoking (p=0.464), or alcohol consumption (p=0.645) (Table 3).

**Table 1:** Patient throat swab completion and antibiotic prescription

	No Throat Swab	Throat Swab
No antibiotic	139 (59.9%)	4 (30.8%)
Antibiotic	93 (40.1%)	9 (69.2%)

**Table 2:** Demographic characteristics of patients with and without throat swab *Missing values not included when calculating column percentages.

Characteristic	All Patients	No Throat Swab	Throat Swab	p-Value
	N (%)*, N = 245	N (%)*, N = 232	N (%)*, N = 13	
Age, mean (SD)	40.2 (16.8)	40.6 (16.8)	32.5 (15.8)	0.093
Sex				0.961
Female	159 (66.0%)	150 (65.8%)	9 (69.2%)	
Male	82 (34.0%)	78 (34.2%)	4 (30.8%)	
Race/ethnicity				0.030
White or Caucasian	62 (36.7%)	60 (38.0%)	2 (18.2%)	
Black or African American	25 (14.8%)	20 (12.7%)	5 (45.5%)	
Asian	18 (10.7%)	17 (10.8%)	1 (9.1%)	
Hispanic, all races	64 (37.9%)	61 (38.6%)	3 (27.3%)	
Employment				0.984
Employed	46 (57.5%)	42 (57.5%)	4 (57.1%)	
Unemployed	34 (42.5%)	31 (42.5%)	3 (42.9%)	

**Table 3:** Prevalence of comorbidities and substance use among patients with symptoms of pharyngitis *Missing values not included when calculating column percentages.

Comorbidity/Substance	All Patients	No Throat Swab	Throat Swab	p-Value
	N (%)*, N = 245	N (%)*, N = 232	N (%)*, N = 13	
Diabetes	33 (13.5%)	31 (13.4%)	2 (15.4%)	0.835
COPD	21 (8.6%)	20 (8.6%)	1 (7.7%)	0.907
Asthma	19 (7.8%)	18 (7.8%)	1 (7.7%)	0.993
Bronchitis	15 (6.1%)	15 (6.5%)	0 (0%)	0.725
Smoking	36 (14.7%)	35 (15.1%)	1 (7.7%)	0.464
Alcohol	28 (11.4%)	26 (11.2%)	2 (15.4%)	0.645

## Discussion

Dedicated efforts toward antibiotic stewardship can reduce unnecessary prescription of antibiotics and decrease healthcare costs and anti-microbial resistance. Pharyngitis in particular provides a useful lens through which prescription practices can be assessed since this pathology can be viral or bacterial in etiology, with only bacterial etiologies warranting antibiotic treatment. Our study contributes to the available body of literature by assessing the current prescription practices of antibiotics in the Tampa Bay Area's uninsured population. Based on our findings, the prescription of antibiotics was associated with the completion of a throat swab. However, of the cases studied, 91.2% of patients received antibiotics for pharyngitis symptoms without a throat swab, revealing that there is still room for provider counseling on current antibiotic prescription recommendations. Currently, the American Academy of Family Physicians (AAFP) recommends clinicians to use a validated clinical decision calculator (e.g., Centor score, McIsaacs score, FeverPAIN score) followed by a RADT if clinical decision-making scores indicate. Antibiotics are then only prescribed given positive RADT or high scores [12]. However, for the purpose of this study, we chose not to use a specific score for identification as McIsaac et al. found that prescribing rates using clinical decision scores did not have a significant difference when compared to using clinical practice [13]. Furthermore, the differing practices of each physician and clinic did not allow for standardization of data collection between all clinics. Additionally, Linder et al. found that clinicians often do not use the scores in their clinical practice [14]. More efforts are needed to use validated clinical decision calculators, which, in turn, can improve antibiotic prescription practices. Proper testing will not only reduce unnecessary prescribing of antibiotics but also prevent widespread antibiotic resistance.

However, compliance with antibiotic prescription clinical guidelines among providers serving the uninsured has not been well-studied in the literature. A study conducted by Sarpong et al. found that uninsured patients use fewer antibiotics than insured patients. They also found that socioeconomic status and racial-ethnic differences can contribute to the difference in prescription practices [15]. This is because uninsured patients, when compared to insured and patients of high socioeconomic status, face more barriers to access to antibiotics. Thus, while antibiotics are over-prescribed on a national level, the uninsured population faces unique struggles regarding prescription practices [16]. Shaver et al. found that uninsured patients were prescribed significantly fewer antibiotics than patients with any private insurance but at a higher rate than patients with public insurance only [17]. Further research on uninsured populations may elucidate this discrepancy between uninsured and insured patients and provide insight into the uninsured population's prescription practices.

One possible reason for the difference in prescription practices of antibiotics to the uninsured is that many uninsured individuals obtain unprescribed antibiotics from other countries or unsound sources within the United States [18-20]. The lack of regulation and control of antibiotics in the uninsured population demonstrates the importance of understanding the practice of prescribing antibiotics within this cohort of patients.

The most notable limitations of our study were the limited sample size and low overall frequency of throat swab completion. Also, our study was limited in that the methodology did not examine the root causes of non-adherent antibiotic prescribing practices such as lack of patient education on antibiotic prescription practices or providers’ concern about the difficulty of following up with patients after test results are returned. It is also possible that scarce financial resources at these free clinics were a barrier to adequate throat swab supplies or that insufficient training resources were made available to promote provider antibiotic stewardship. Nevertheless, our study illustrates that free clinics need more support to be able to follow appropriate screening guidelines.

## Conclusions

Our study found that only a small percentage of patients who were prescribed an antibiotic completed a throat swab, inversely correlating antibiotic prescription with a throat swab. The uncommon utilization of throat swabs suggests a need for increased support of free clinics to improve prescription practices. Although these results suggest an inverse relationship between antibiotic prescription and throat swabs, more research should be conducted to assess other factors that may be contributing to antibiotic over-prescription and specific gaps in the uninsured population that create obstacles for the appropriate treatment of pharyngitis in this vulnerable population.
